# Low-Density Granulocytes in Immune-Mediated Inflammatory Diseases

**DOI:** 10.1155/2022/1622160

**Published:** 2022-01-31

**Authors:** Xin Ning, Wen-Ming Wang, Hong-Zhong Jin

**Affiliations:** Department of Dermatology, State Key Laboratory of Complex Severe and Rare Diseases, Peking Union Medical College Hospital, Chinese Academy of Medical Science and Peking Union Medical College, National Clinical Research Center for Dermatologic and Immunologic Diseases, Beijing, China 100730

## Abstract

Low-density granulocytes (LDGs), a distinct subset of neutrophils that colocalize with peripheral blood mononuclear cells after density gradient centrifugation, have been observed in many immune-mediated diseases. LDGs are considered highly proinflammatory because of enhanced spontaneous formation of neutrophil extracellular traps, endothelial toxicity, and cytokine production. Concomitantly, increased numbers of LDGs are associated with the severity of many immune-mediated inflammatory diseases. Recent studies, with the help of advanced transcriptomic technologies, demonstrated that LDGs were a mixed cell population composed of immature subset and mature subset, and these two subsets showed different pathogenic features. In this review, we summarize the current knowledge on the composition, origin, and pathogenic properties of LDGs in several immune-mediated inflammatory diseases and discuss potential medical interventions targeting LDGs.

## 1. Introduction

Neutrophils are key players in the innate immune system, and they are one of the first responders against infectious agents and other pathogenic assaults [1, 2]. Recently, neutrophil dysregulation was reported to have a critical role in various immune-mediated inflammatory diseases [3]. Originally, neutrophils were considered a homogeneous group of white blood cells; however, recent studies revealed the heterogeneity of neutrophil populations with regard to their morphology, phenotype, and function [4–6]. Low-density neutrophils (LDNs) represent a distinct subpopulation of neutrophils of increasing interest due to their unusual intrinsic properties and their upregulation in various immune-related abnormalities, infectious diseases, and malignancies [6–9]. LDNs were first identified in systemic lupus erythematosus (SLE) patients in 1986. Because of their relative low density (<1.077 g/ml), LDNs cosegregate with peripheral blood mononuclear cell (PBMC) fraction after Ficoll gradient centrifugation, while most neutrophils sediment within the erythrocyte fraction (often termed normal-density neutrophils (NDNs)) [10]. The phenotype of LDNs may vary among different disease backgrounds. In several malignancies and inflammatory diseases, LDNs exhibit immunosuppressive properties (also termed granulocyte myeloid-derived suppressor cells) [3, 5, 11]. Whereas, in many immune-mediated diseases, LDNs present with highly proinflammatory properties and have been termed low-density granulocytes (LDGs) by many researchers [11, 12]. Here, we focus on the proinflammatory profile of LDGs.

Compared with NDNs, LDGs exert an enhanced ability to generate neutrophil extracellular traps (NETs) spontaneously and synthesize high amounts of proinflammatory cytokines (e.g., type I interferons (type I IFNs), TNF-*α*) [11, 13–15]. Thus, LDGs can contribute to the pathogenesis of various immune-mediated inflammatory diseases (e.g., SLE, psoriasis, and adult-onset Still's disease (AOSD)), which is in line with the findings that the amount of LDGs correlates with the severity of immune disorders [12, 16, 17]. Therefore, targeting LDGs might be a promising therapeutic approach to alleviate certain immune dysregulations.

Currently, the composition, origin, and pathogenic mechanisms of LDGs are still controversial. This review will focus on recent studies related to the understanding of the nature of LDGs and promising LDGs-related treatment methods in immune-mediated inflammatory diseases.

## 2. Heterogeneity and Origins of LDGs

### 2.1. Evidence Supporting the Immaturity of LDGs

Morphologically, LDGs were reported to contain segmented, banded, and myelocyte-like nuclei [14, 18, 19]. Ultrastructural analysis based on transmission electron microscopy revealed that LDGs generally have less segmented nuclei compared with NDNs [6]. In addition, the proportion of cells with immature nuclear morphology in LDGs was higher than that in NDNs (e.g., 40% vs. 10% in SLE) [14]. Therefore, the nuclear morphology of LDGs indicates a lower grade of maturity in contrast to NDNs. Moreover, genomic analyses showed that the LDG gene signature was clustered in granule protein synthesis and cell cycle regulation, which is typical of the early stages of neutrophil development [16, 20]. Indeed, neutrophil progenitors were reported to have a low density, which increased gradually during granulocyte maturation [21]. Based on the features described above, it has been postulated that LDGs are prematurely released by neutrophil progenitors from the bone marrow [6]. During inflammatory conditions, various stimuli (e.g., granulocyte colony-stimulating factor (G-CSF), granulocyte-macrophage colony-stimulating factor (GM-CSF), and type I IFNs) elicit augmented granulopoiesis leading to increased numbers of immature low-density neutrophils that are segmented to the PBMC fraction [18, 22]. Hassani et al., used lipopolysaccharide (LPS) 2 ng/kg to mimic acute systemic inflammatory conditions in healthy volunteers [22]. After the LPS stimulus, more LDGs were isolated in PBMC fraction after Ficoll gradient centrifugation. Correspondingly, the percentage of neutrophils with an immature phenotype (CD16^dim^/CD62L^high^) in LDGs was significantly increased [22].

### 2.2. Evidence Supporting a More Mature Stage of LDGs

However, the cell surface markers of LDGs indicate a mature phenotype. Previous flow cytometry (FACS) studies showed that LDGs express molecular markers typically expressed by mature neutrophils, including CD10, CD15, and CD16 [14]. Herteman et al. measured intracellular myeloperoxidase (MPO) levels and N-formylmethionine-leucyl-phenylalanine receptor (fMLP-R), which is synthesized at the terminal stage of granulocyte maturation, as indicators of LDG maturity in healthy and asthmatic horses. The results indicated a mature stage because LDGs contained a comparable level of MPO and higher level of fMLP-R relative to NDNs [11]. Comparable expression of MPO and fMLP-R between LDGs and NDNs has also been observed in SLE patients and psoriasis patients, respectively [10, 15]. Compared with neutrophils isolated from healthy controls (HCs), LDGs in SLE and advanced lung adenocarcinoma patients exhibit a more activated phenotype due to high CD66b and CD11b expression on the cell surface [14, 23]. Therefore, it is supposed that LDGs arise from the activation of NDNs. In favor of this, Hassani et al. activated neutrophils from healthy donors with fMLP and platelet activating factor (PAF) *in vitro*, which decreased the neutrophil density. After stimulation, LDGs consistently displayed a higher expression of activation markers (CD66b, CD11b, CD35, and CD16) and lower CD62L compared with the top 20% of the highest density neutrophils [22]. This suggests that a portion of mature NDNs may be more sensitive to proinflammatory stimuli and that they tend to be distributed in the PBMC density fraction after activation. The potential mechanism of this neutrophil density shift may be related to degranulation [24]. However, a recent electron microscopy study did not support the degranulation hypothesis [6]. Alternatively, the decrease in neutrophil density might result from water influx mediated by aquaporin-9 [25]. Recently, Su et al. reported that *Mycobacterium tuberculosis* infection induced the generation of LDGs from NDNs by a mechanism involving reactive oxygen species- (ROS-) dependent NET formation [26]. Furthermore, it has been proposed that LDGs originated from a distinct neutrophil lineage as a result of genomic abnormalities, including increased copy number alterations, losses of heterozygosity, and microsatellite instability [27]. Based on the aforementioned facts, the nuclear morphology and gene expression profile of LDGs is inconsistent with their surface marker phenotype with regard to cell maturity, which suggests that LDGs might not be exclusively composed of mature or immature cells.

### 2.3. LDGs Are Mixed Cell Population

Recently, an increasing number of studies have suggested that LDGs represent a heterogeneous population composed of mature and immature neutrophils [18, 19]. This model is helpful to resolve the discord among LDGs' nuclear morphology, gene expression profile, and surface marker pattern. Immature LDGs are more transcriptionally active and contribute to the granulopoiesis gene signature in the bulk transcriptomic data of LDGs, while mature LDGs, accounting for the majority of LDGs, shape the surface marker phenotype of total LDG population. Mistry et al. divided LDGs from SLE patients into two groups based on the expression of CD10, which is only expressed in the final stage of neutrophil development [19, 28]. CD10^−^ LDGs resemble neutrophil precursors with less segmented nuclei, higher transcriptional activity, and enhanced cell cycle gene expression. In contrast, CD10^+^ LDGs showed multilobulated nuclei, relatively repressed transcriptional activity, and elevated expression of type I IFN-stimulated genes (ISGs). Compared with CD10^−^ LDGs, CD10^+^ LDGs perform enhanced canonical neutrophil functions including chemotaxis, NET formation, and phagocytosis but impaired degranulation [19]. Thus, CD10^+^ LDGs displayed a relatively mature stage of neutrophil development. Of note, CD10^+^ LDGs cannot be defined as fully matured neutrophils because they displayed higher expression of cell cycle genes and transcription factors (e.g., *CEBPA* and *IRF8 [*2*]*) that are suggestive of immature neutrophils compared with CD10^+^ NDNs [19]. Therefore, it remains unclear whether the intermediate-mature CD10^+^ LDGs subset is an extension of a continuous spectrum of activated NDNs, or an abnormally developed neutrophil subset distinct from autologous NDNs. The origin and composition of LDGs may vary with different diseases and different pathological stages [5]. Anyway, the currently known characterizations of LDGs under various conditions are collectively discussed below.

## 3. Role of LDGs in Immune-Mediated Inflammatory Diseases

The number of circulating LDGs increased significantly in SLE, psoriasis, idiopathic inflammatory myopathies (IIM), adult-onset Still's disease (AOSD), and other immune-mediated inflammatory disorders and has been found to be associated with disease severity. Apart from clinical quantitative measurements, the functional properties of LDGs have also been investigated in several diseases. Denny et al. reported that LDGs in SLE patients had an augmented capacity to damage endothelial cells and synthesize proinflammatory cytokines (e.g. IFN-*α*, TNF-*α*, and IFN-*γ*) compared with autologous NDNs from lupus patients and HCs [14]. LDGs also show enhanced spontaneous NET formation compared with NDNs in SLE and other immune-related diseases [13, 15, 26, 29]. However, Wright et al. investigated the functional properties of LDGs from patients with rheumatoid arthritis (RA) and found that their cytokine expression, ROS production, NET formation, chemotaxis, and phagocytosis, but not cell survival, were inferior to RA neutrophils [30]. These functional discrepancies indicate the diversity of LDG origins among different diseases. Therefore, the roles of LDGs in related immune-mediated inflammatory diseases will be discussed separately in the following sections.

### 3.1. Systemic Lupus Erythematosus (SLE)

SLE is an autoimmune disease featured by multiorgan damages and prominent abnormality of innate and adaptive immune systems [31]. Previous studies have reported the augmentation of LDGs in SLE patients [6, 10] and that the circulating LDG count is strongly associated with SLE activity measured by the SLE disease activity index (SLEDAI) scores [12, 32]. In addition, Rahman et al. reported that the LDG-to-lymphocyte ratio (LLR) was a more sensitive indicator of SLE activity than the neutrophil-to-lymphocyte ratio, because LLR provided a better resolution between inactive SLE patients and healthy controls [12]. Kegerreis et al. identified an LDG-specific gene module enriched in the peripheral blood that correlated with increased SLEDAI as well as low serum complement levels, anti-dsDNA seropositivity, and corticosteroid administration [20].

Potential mechanisms related to the pathogenic effects mediated by LDGs in SLE include increased synthesis of proinflammatory cytokines and NET formation [13, 14]. Denny et al. reported that lupus LDGs express a higher level of IFN-*α* mRNA and secrete increased TNF-*α* and IFN-*γ* upon phorbol-12-myristate-13-acetate (PMA) stimulation when compared to autologous lupus NDNs and NDNs from HCs [14]. Type I IFNs, which include five classes (IFN-*α*, IFN-*β*, IFN-*ω*, IFN-*ε*, and IFN-*κ*), play a key role in human antiviral immunity. But type I IFNs are also causative factors of autoimmune diseases [33]. Type I IFNs can evoke general mobilization of immune cells, including dendritic cells, Th1 cells, cytotoxic T cells, and B cells, and induce persistent inflammation and tissue damage [33]. LDGs are also key responders to type I IFNs. Rahman et al. observed an association between a high ISGs signature and increased LDG frequency [12].

Enhanced spontaneous NET formation is another well-known proinflammatory mechanism of LDGs in SLE. NETs are reticular structures that consist of chromatin, granule proteins, and cytosolic contents [34, 35] and function as an antimicrobial mechanism to restrain and kill microbes; however, the altered formation and removal of NETs can be detrimental in SLE [35]. LL-37, an antimicrobial peptide in NETs, combines with DNA to trigger type I IFNs production in plasmacytoid dendritic cells via Toll-like receptor 7 (TLR7) and TLR9 signaling [35, 36]. Furthermore, type I IFNs induce neutrophils to release NETs resulting in a self-lasting loop of pathogenic inflammation [6]. NETs display antigenic components (e.g., double-stranded deoxyribonucleic acid (DNA), oxidized mitochondrial DNA, and citrullinated proteins) that might participate in the generation of autoantibodies [35]. The enhanced spontaneous NET formation in SLE LDGs may be related to mitochondrial reactive oxygen species (mtROS). LDGs in SLE produce excessive mtROS, and the scavenging mtROS significantly suppressed NET formation in SLE LDGs [37].

Of note, type I IFNs and NETs are causative agents of vascular damage, indicating the pathogenic role of LDGs in vascular comorbidity in SLE. Type I IFNs induced apoptosis in endothelial cells and impaired the maturation of endothelial progenitors [4]. NETs released from LDGs elicited vascular injury probably by inducing endothelial cytotoxicity and providing a scaffold for thrombosis [13, 16, 35]. Furthermore, SLE LDGs showed increased retention in the microvasculature compared with autologous NDNs and may contribute to the excessive small vessel vasculopathy in SLE [38]. Calrucci et al. reported that LDGs expressed a higher level of genes correlated with vascular inflammation and the noncalcified coronary plaque burden (NCB) in SLE patients, compared to NDNs [39]. Recently, López et al. demonstrated that the ratio of CD16^−^/CD14^−^ LDGs to high-density lipoprotein-cholesterol helped identify cardiovascular disease (CVD) risks [40]. Jointly, these findings suggest that LDGs have a key role in lupus activity and corresponding vascular damage. However, there is still a lack of direct evidence for the role of LDGs in other lupus-induced organ damages, such as lupus nephritis.

As described above, LDGs from SLE patients consist of two subsets including CD10^+^ intermediate-mature LDGs and CD10^−^ immature LDGs. Mistry et al. found that CD10^+^ LDGs had the highest level of ISGs expression among various myeloid subtypes in PBMCs and showed enhanced the ability of spontaneous NET formation compared with CD10^−^ LDGs [16]. Furthermore, the number of CD10^+^ LDGs positively correlated with the NCB severity and negatively correlated with the cholesterol efflux capacity (a protective indicator of cardiovascular events), suggesting a vasculopathic role of CD10^+^ LDGs in SLE. The CD10^−^ immature LDGs may contribute to immune dysregulation through other mechanisms. Marini et al. found that CD10^−^ LDGs from G-CSF-treated donors promote the survival, proliferation, and IFN-*γ* production of T cells, whereas CD10^+^ LDGs showed opposite functions [18]. Similarly, SLE LDGs do not restrain T cell proliferation but promote the production of IFN-*γ*, TNF-*α*, and lymphotoxin-*α* from CD4^+^ T cells, which may be due to the relatively high proportion of CD10^−^ immature cells in SLE LDGs [12]. More elaborate subdivision of LDGs, based on immunophenotype or other markers, might help to elucidate the pathogenesis of SLE and other inflammatory disorders.

### 3.2. Psoriasis

Psoriasis is a chronic inflammatory disease mainly presenting with skin symptoms characterized by scaly erythema, papules, and plaques [41]. Patients with psoriasis are also susceptible to early-onset atherosclerosis and ensuing cardiovascular complications [42]. Neutrophils have been extensively studied in the pathogenesis of psoriasis [36, 43]. However, few studies have investigated the role of LDGs in psoriasis. We explored the gene expression profiles in PBMCs from patients with generalized pustular psoriasis (GPP) and found a marked enrichment of differentially expressed genes related to neutrophil functions, implicating the presence of LDGs in GPP PBMCs [44]. Teague et al. reported the number of LDGs was positively associated with psoriasis severity, which was measured by the psoriasis area and severity index (PASI) [16].

Like lupus LDGs, psoriasis LDGs have been reported to show augmented NET formation activity, which elucidate, if not all, at least part of their proinflammatory mechanism in psoriasis. NETs play a key role in the initial and maintenance stages of psoriasis. Targeting NETs with DNase I or CI-amidine *in vivo* alleviated disease severity in an imiquimod-induced psoriasis-like mouse model [45]. By externalizing immunogenic molecules (e.g., S100A8, S100A9, lipocalin 2, *β*-defensin, LL-37, and heat shock proteins 70), NETs induce a highly proinflammatory microenvironment [36, 46–49]. NETs also activate keratinocytes to produce inflammatory cytokines (e.g., IL-36*γ* and lipocalin 2) via TLR4-IL36 receptor cross talk [45]. Moreover, IL-17 and IL-36, two important psoriasis-related cytokines, have close links with NETs [50–52]. Lin et al. reported that LDGs released IL-17 via NET formation in psoriasis [53]. Furthermore, Th17 cells can be induced from human PBMCs by NETs with the assistance of monocytes *in vitro* [54]. In addition, serine proteases displayed by NETs (e.g., proteinase 3 and neutrophil elastase) can cleave pro-IL-36 to improve IL-36 activity [55–57].

Several studies have investigated the mechanism by which LDGs promote NETs in psoriasis. Skrzeczynska-Moncznik et al. reported that LDGs and NDNs show difference in the ability to extrude NETs because of their distinct levels of free neutrophil elastase (NE) which is crucial for NET formation. They investigated LDGs and NDNs in psoriasis and found that LDGs had increased staining for NE and reduced staining for secretory leukocyte protease inhibitor (SLPI), an NE inhibitor [15]. LDGs also showed greater chemotactic migration to psoriasis cutaneous extracts compared with NDNs [15]. These distinct staining patterns suggest the different cytosolic distribution of these two proteins, which is likely to be reflected in the heterogeneity of NETs production and chemotaxis in LDGs and NDNs [15]. Another potential mechanism of NET formation involves platelet-LDG interactions. Platelets have been reported to promote neutrophils to release NETs in sepsis and acute lung injury [58–60]. A study by Teague et al. showed psoriasis LDGs upregulated the transcription of genes related to platelet-LDG aggregation, and electron microscope analysis indicated that platelet adherence only occurred on LDGs but not NDNs [16]. Moreover, the proportion of LDGs spontaneously releasing NETs correlated with the circulating platelet frequency in psoriasis, suggesting the potential effect of platelet aggregation in NET formation of LDGs. In line with the atherogenic role of NETs, LDGs aggregated with platelets showed a linear correlation with early NCB in psoriasis [16]. In consensus with the atherogenic role of NETs, LDGs aggregated with platelets showed a linear correlation with early NCB in psoriasis [16].

### 3.3. Antineutrophil Cytoplasmic Autoantibody- (ANCA-) Associated Vasculitis (AAV)

AAV is a systemic autoimmune disease where autoantibodies target MPO and proteinase 3(PR3). Ui Mhaonaigh et al. reported that the proportion of LDGs among total granulocytes in active AAV was three times higher than that in remission or controls [61]. In addition, the gene expression of MPO and PR3 was augmented in LDGs from patients with active AAV compared with HCs. However, LDGs were insensitive to anti-MPO antibodies in contrast to NDNs [61, 62]. Therefore, LDGs might not mediate vascular damage via ANCA-induced ROS production. The escalation of LDGs is more likely an outcome of increased granulopoiesis in acute inflammation, since the transcription of azurophilic granular proteins, including MPO and PR3, mainly occurs in the promyelocytic stage of neutrophil differentiation. Although LDGs are hyporesponsive to MPO-ANCA stimulation, LDGs externalizing MPO and PR3 by enhanced NET formation have been isolated from the PBMCs of AAV patients and might provide a source of autoantigens [63].

### 3.4. Adult-Onset Still's Disease (AOSD)

AOSD is a systemic autoinflammatory disease characterized by intermittent fever, rash, arthritis, and neutrophilia [64]. Several proinflammatory cytokines, including IL-6, TNF-*α*, and IL-18, play key roles in the pathogenesis of AOSD [65]. Recently, Liu et al. reported that the number of circulating LDGs significantly correlated with C-reactive protein, erythrocyte sedimentation rate, and the modified Pouchot score in AOSD patients [17]. They also found that LDGs were another critical source of IL-6 in addition to CD14^+^ monocytes and therefore might be one of the key drivers of AOSD [17]. G-CSF, a principal inducer of granulocytic hyperplasia, was increased in AOSD patients and was positively associated with circulating LDGs counts, supporting the hypothesis that LDGs are neutrophil precursors prematurely mobilized from the bone marrow by G-CSF stimulation [66].

### 3.5. Graft-versus-Host Disease (GVHD)

GVHD is a multiorgan-involved complication of allogeneic haematopoietic stem cell transplantation and can be divided into acute GVHD (aGVHD) and chronic GVHD (cGVHD) by onset time and clinical manifestations [67, 68]. LDGs were initially found to be increased by extracorporeal photopheresis or G-CSF treatment and suppressed Th1 and Th17 response in aGVHD [69]. However, recently in cGVHD, LDGs have been discovered to be proinflammatory, since they promote T cell proliferation and IFN-*γ* and IL-6 production and prove high resistance against constitutive apoptosis [70]. This is probably due to the high proportion of CD10^−^ immature LDGs (mean value and interquartile range: 39% and 17–76%), which were previously found to enhance T cell proliferation and IFN-*γ* production in G-CSF-treated donors, among cGVHD LDGs [70]. It is noted that, under the exposure to granulocyte-macrophage colony-stimulating factor (GM-CSF) and IFN-*γ*, the predominantly CD10^−^ LDGs from cGVHD patients can even differentiate into antigen-presenting/neutrophil-hybrid-like cells, which express major histocompatibility complex class II [70]. These data suggest that LDGs may have pathogenic potential in cGVHD.

### 3.6. Coronavirus Disease 2019 (COVID-19)

COVID-19, which is caused by SARS coronavirus 2 (SARS-CoV-2) infection, has become a pandemic of worldwide concern. The clinical manifestations of COVID-19 range from asymptomatic or mild upper-respiratory tract infection to severe lower-respiratory tract disease which may progress to life-threatening acute respiratory distress syndrome [71]. Patients with COVID-19 are also susceptible to venous and arterial thrombosis [72, 73] and demonstrate features of autoimmunity [74, 75]. Frequency of LDGs increases significantly in COVID-19 patients and correlates with disease severity [76–78], supporting earlier transcriptomic data disclosing the presence of neutrophils in PBMC from COVID-19 patients [79, 80]. LDGs have been found to suppress the proliferation of autologous T cells in *ex vivo* assays and may contribute to lymphopenia and impaired immune response during severe SARS-CoV-2 infection [77, 78, 80]. Nonetheless, LDGs from severe COVID-19 patients, especially LDGs with intermediate CD16 expression (CD16^int^ LDGs), have been found to be prone to form NETs and aggregate with platelets, which promote alveolar cell apoptosis and thrombogenic coagulopathy [81, 82]. Moreover, anti-NET antibodies have been identified in severe COVID-19 patients and display correlation with antinuclear antibody and ANCA positivity, suggesting autoimmune response induced by NETs [81]. These finds indicate that LDGs impede antiviral responses of T lymphocytes but boost aberrant hyperinflammation and autoimmunity in severe COVID-19 patients.

### 3.7. Other Immune-Mediated Disorders

Low-density neutrophils or LDG gene signatures have also been observed in many other immune-mediated disorders, including IIM, RA, juvenile idiopathic arthritis, and Pyogenic arthritis, pyoderma gangrenosum, and acne (PAPA) syndrome [9, 30, 65, 83–86]. The classical enhancement of spontaneous NET formation has been observed in LDGs from IIM, PAPA syndrome, and equine asthma. Of note, RA LDGs showed deficiencies in NET formation, as well as cytokine synthesis, phagocytosis, and chemotaxis. The apoptosis rate and response to TNF-*α* were decreased in RA LDGs, which may facilitate drug resistance to TNF-inhibitors [30]. Therefore, low-density neutrophils might represent disparate neutrophil subsets in different diseases and have different pathogenic properties. The immunophenotypes and functional properties of LDGs in various immune-mediated inflammatory diseases are summarized in Table 1.

## 4. Therapeutic Methods Targeting LDGs

Targeting the key nodes in the procedure by which LDGs playing their proinflammatory roles may suggest novel therapeutic methods for immune-mediated inflammatory diseases. These critical nodes include the production, migration, and NET formation of LDGs. GM-CSF and G-CSF, which activate the production of neutrophils and induce the increase of LDGs, are potential treatment targets for restricting the quantity of LDGs [87, 88]. Inhibiting crucial chemoattractants and chemokine receptors for neutrophil mobilization, such as C-X-C motif ligand 2 (CXCL2), CXCL8 [89, 90], CXC chemokine receptor 1 (CXCR1)/CXCR2 [91, 92], and fMLP-R, could help to decrease the LDGs infiltration in peripheral tissues.

NET formation, which leads to the exposure of autoantigens, damage to tissues, interferon production, and activation of immune cells, is also a prospective target for developing therapeutic methods to prevent LDG-mediated inflammation. Immune complexes, complement 5a [93], type I IFNs [94], and activated platelets [95] have been reported to induce the release of NETs, suggesting the potential of strategies targeting these triggers. It has also been suggested that mtROS play a key role in the increase of NET formation of SLE LDGs. Agents inhibiting mtROS, including idebenone and MitoTEMPO, have been proved to suppress spontaneous NET formation of SLE LDGs and reduce disease activity in murine models of lupus [37]. Peptidylarginine deiminase 4 (PAD4), which facilitates chromatin decondensation during NET formation [96], has become a potential therapeutic in recent years. PAD4 inhibitors, such as chloramidine, have shown efficacy in SLE and psoriasis models [45, 97]. However, a complete block of PAD4 may remarkably increase the risk of infection. Apart from restraining NET formation, disrupting NETs structure directly with deoxyribonuclease I (DNAse I) has proven efficient in alleviating NET-induced inflammation [98]. Hydroxychloroquine, a clinically widely used drug to treat SLE, has been found to inhibit NET formation via blocking TLR9 [99]. In addition, Janus kinase (JAK) inhibitors, such as tofacitinib (JAK1/3), are capable of inhibiting migration and NET formation of NDNs [100, 101] and may also be potent in LDGs. Taken together, targeting LDGs could provide novel entry points to treat immune-mediated inflammatory diseases (Table 2).

## 5. Conclusions and Perspectives

**Figure 1:**
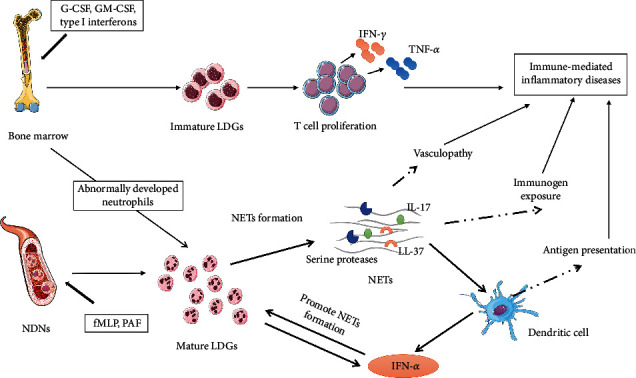
The composition, origin, and pathogenic properties of LDGs. LDGs represent a heterogeneous cell population containing an immature subset and a mature subset. The immature subset may result from increased granulopoiesis in acute inflammation, while the mature subset may arise from activated NDNs or aberrant neutrophil precursors. These LDGs subsets might be involved in the pathogenesis of immune-mediated inflammatory diseases by different mechanisms. LDGs; low-density granulocytes; NDNs: normal-density neutrophils; NETs: neutrophil extracellular traps; fMLP: N-formylmethionine-leucyl-phenylalanine; PAF: platelet activating factor. Elements in this figure are provided by Servier Medical Art [108].

The elevation of circulating LDGs has been observed in a variety of immune-mediated inflammatory diseases and may implicate a novel pathogenic mechanism in immune-mediated diseases. LDGs were initially regarded as a homogeneous cell population integral, while recent studies noted the heterogeneity in LDGs. Based on studies to date, we hypothesize that LDGs contain two subsets—an activated mature subset and an immature subset (Figure 1). The activated mature subset might result from the activation of NDNs, whereas the immature subset might be derived from enhanced granulopoiesis in the context of systemic inflammation. The mature LDG subset displays better canonical neutrophil pathogenic functions, such as NET formation, compared with the immature subset, while the later subset facilitate the proliferation and cytokine production of T lymphocytes.

Nonetheless, many issues about LDGs remain to be elucidated. The actual composition and origins of LDG populations might be more complicated than what the current data suggest, hence the need for further investigations to precisely classify and characterize each LDG subset. Functional and transcriptional analyses suggest that CD10^+^ mature LDGs are at a lower maturity level compared with common NDNs [19, 61]. Thus, CD10^+^ LDGs may be derived not simply from the activation of NDNs, but from some abnormally developed neutrophils. Another unsolved issue is whether LDGs are indeed associated with CVD risks in patients with autoimmune or inflammatory diseases. Although LDGs are correlated with some risk factors of CVD in several cross-sectional or retrospective studies, the longitudinal data demonstrating the relation between LDGs and cardiovascular events is still unavailable [16, 39, 40]. The direct proof of LDGs infiltration in organ and tissue involvements remains absent as well [6, 20]. Moreover, how LDGs acquire enhanced proinflammatory properties remains to be elucidated. Once these questions are clarified, we may be able to develop measures to attenuate the pathogenicity or diminish the generation of LDGs.

In a word, this review discussed recent advancement in the investigation of characteristics and functions of LDGs in immune-mediated inflammatory diseases. Further investigation for LDGs might lead to better insights into the pathogenesis of immune-mediated disorders and enable the development of novel therapeutic methods targeting LDGs.

## Figures and Tables

**Table 1:** Immunophenotypes and functional properties of LDGs in immune-mediated inflammatory diseases.

Diseases	References	Surface markers	Status	Functional properties
SLE	[13, 14]	CD10^+^ CD11c^low^ CD14^low^ CD15^high^ CD16^high^ CD31^+^ CD114^+^ CD116^−^ cells	Heterogeneous population of mature and immature cells	Enhanced synthesis of IFN-*α* and TNF-*α*; enhanced spontaneous NET formation and IL-17 externalization; impaired phagocytosis
	[37]	ND	ND	Enhanced production of mtROS and NETs
	[19]	CD10^−^	Immature cells with less segmented and more rounded nuclei	Enhanced degranulation; impaired NET formation, phagocytosis, chemotaxis
		CD10^+^	Intermediate-mature cells with multilobulated nuclei	High expression of ISGs; enhanced spontaneous NET formation, oxidized mit-DNA secretion, endothelial cytotoxicity
	[12]	LOX-1^high^ CD63^high^ CD107a^high^ CD274^high^ Arg1^low^ CD273^low^ CD95^low^	Activated; heterogeneous population of mature and immature cells	Promoting of Th1 cytokine production (including IFN-*γ*, TNF-*α*, and lymphotoxin alpha)

Psoriasis	[53]	CD10^+^ CD14^low^ CD15^+^	ND	Enhanced spontaneous and PMA-induced NET formation
	[15]	CD10^+^ CD11b^+^ CD15^high^ CD66b^high^ CD62L^low^	A majority of mature cells and a minority of immature cells	Enhanced spontaneous NET formation; elevated tendency to migrate to psoriasis skin extracts
	[16]	CD14^low^ CD15^high^ CD10^high^	ND	Enhanced spontaneous NET formation; endothelial cytotoxicity; enhanced neutrophil-platelet aggregates

RA	[30]	CD10^+^ CD14^+^ CD15^high^	ND	Impaired chemotaxis, phagocytosis, and PMA-induced NET formation; hyporesponsive to TNF-*α*; enhanced cell survival

AAV	[63]	ND	ND	Enhanced spontaneous NET formation
	[61]	CD16^+^ CD10^+^ LOX-1^low^ CD88^+^ CD177^+^	Mature cells with multilobulated nuclei	Hyporesponsive to anti-MPO antibody
		CD16^int/-^ CD10^−^ LOX-1^high^ CD88^low^ CD177^low^	Immature cells with circular or kidney-shaped nuclei	Unresponsive to anti-MPO antibody
	[62]	CD10^+/-^ CD15^+^	Heterogeneous of mature subset and immature cells	Elevated autoantigen gene expression; unresponsive to anti-MPO antibody

AOSD	[17]	CD10^-/+^ CD14^-/low^ CD15^+^	ND	Enhanced contribution to IL-6 production

PAPA syndrome	[83]	CD10^−/+^ CD14^low^/CD15^+^	ND	Enhanced spontaneous NET formation

IIM	[84]	CD10^+^ CD15^+^ CD14^low^	ND	Enhanced spontaneous NET formation

cGVHD	[70]	Predominantly CD10^–^ CD16^–/dim^ CD11b^dim/high^ LOX-1^+^	Immature	Promoting T cell proliferation and IFN-*γ* and IL-6 production, resisting consecutive apoptosis, having the potential to differentiate into APC-N hybrids

COVID-19	[77]	CD16^−^ CD11b^−^, CD16^−^ CD11b^+^	Immature	Reflecting the emergency myelopoiesis
		CD16^+^ CD11b^+^	Intermediate mature	May contribute to immunosuppression of T cell and NET formation
		CD16^+^ CD11b^-/low^	Mature	
	[76]	CD16^Int^ CD11b^Int^ CD40^+^	Immature	Exhibiting proinflammatory gene signatures with increased phagocytic capacity, spontaneously forming NETs, activating platelets

ND: not defined, SLE: systemic lupus erythematosus, PMA: phorbol-12-myristate-13-acetate, mtROS: mitochondrial ROS, RA: rheumatoid arthritis, AAV: antineutrophil cytoplasmic autoantibody (ANCA) vasculitis, AOSD: adult-onset Still's disease, PAPA: pyogenic arthritis, pyoderma gangrenosum, and acne, IIM: idiopathic inflammatory myopathies, cGVHD: chronic Graft-versus-host disease, COVID-19: Coronavirus Disease 2019.

**Table 2:** Promising therapies targeting LDGs for immune-mediated diseases.

Target	Mechanism	Medication examples
Production of neutrophils	Inhibiting GM-CSF receptor alpha chain	Mavrilimumab [87]
	Inhibiting JAK1/STAT3 pathway	Tofacitinib, tofacitinib [100]

Migration of neutrophils	Inhibiting IL-8 -CXCR1/CXCR2 signal	DF 2162 [91], RIST4721 [102], HuMab 10F8 [90]

Formation of NETs	Eliminating mtROS	MitoTEMPO [37], Idebenone [103]
	Inhibiting C5a signal	Eculizumab [104], Avacopan [105]
	Inhibiting type I IFNs signal	Tofacitinib [101]
	Reducing autoantibodies and immune complexes	Rituximab and Belimumab [106, 107]
	Inhibiting PAD4 and Rac2 expression by blocking TLR9	Hydroxychloroquine [99]
	Enhancing the degradation of NETs	DNAse I [45, 98]

GM-CSF: granulocyte-macrophage colony-stimulating factor, mtROS: mitochondrial ROS, PAD4: peptidylarginine deiminase 4, DNAse I: deoxyribonuclease I.
